# Depletion of Regulatory T Lymphocytes Reverses the Imbalance between Pro- and Anti-Tumor Immunities via Enhancing Antigen-Specific T Cell Immune Responses

**DOI:** 10.1371/journal.pone.0047190

**Published:** 2012-10-17

**Authors:** Yu-Li Chen, Ming-Cheng Chang, Chi-An Chen, Han-Wei Lin, Wen-Fang Cheng, Chung-Liang Chien

**Affiliations:** 1 Graduate Institute of Anatomy and Cell Biology, College of Medicine, National Taiwan University, Taipei, Taiwan; 2 Gynecologic Cancer Center, Department of Obstetrics and Gynecology, Cathay General Hospital, Taipei, Taiwan; 3 Department of Obstetrics and Gynecology, College of Medicine, National Taiwan University, Taipei, Taiwan; 4 Graduate Institute of Oncology, College of Medicine, National Taiwan University, Taipei, Taiwan; 5 Graduate Institute of Clinical Medicine, College of Medicine, National Taiwan University, Taipei, Taiwan; 6 Department of Anesthesiology, College of Medicine, National Taiwan University, Taipei, Taiwan; University of Southern California, United States of America

## Abstract

**Background:**

The regulatory T cells (Tregs) can actively suppress the immune responses. However, literature about detailed changes of host effective and suppressive immunities before and after depletion of Tregs in ovarian carcinomas, is rare.

**Materials and Methods:**

Ovarian cancer patients and the ascitogenic animal model were employed. Immunologic profiles with flow cytometric analyses, immunohistochemistric staining, RT-PCR, ELISA, and ELISPOT assays were performed. *In vivo* depletion of Treg cells with the mAb PC61was also performed in the animal model.

**Results:**

The cytokines, including IL-4 (*p* = 0.017) and TNF-α (*p* = 0.046), significantly decreased while others such as TGF-β (*p* = 0.013), IL-6 (*p* = 0.016), and IL-10 (*p* = 0.018) were elevated in ascites of ovarian cancer patients, when the disease progressed to advanced stages. The ratio of CD8^+^ T cell/Treg cell in ascites was also lower in advanced diseases than in early diseases (advanced 7.37±0.64 vs. early 14.25±3.11, *p* = 0.037). The kinetic low-dose CD25 Ab depletion group had significantly lower intra-peritoneal tumor weight (0.20±0.03 g) than the sequential high-dose (0.69±0.06 g) and sequential low-dose (0.67±0.07 g) CD25 Ab deletion groups (*p* = 0.001) after 49 days of tumor challenge in the animal. The kinetic low-dose CD25 Ab depletion group generated the highest number of IFN-γ-secreting, mesothelin-specific T lymphocytes compared to the other groups (*p*<0.001).

**Conclusions:**

The imbalance between effective and suppressive immunities becomes more severe as a tumor progresses. The depletion of Treg cells can correct the imbalance of immunologic profiles and generate potent anti-tumor effects. Targeting Treg cells can be a new strategy for the immunotherapy of ovarian carcinoma.

## Introduction

Malignancy is considered a multi-factorial disease and the influence of immunologic mechanisms on cancer progression and prognosis has become an important issue recently. The CD25^+^CD4^+^ regulatory T cells (Tregs) actively suppress physiologic and pathologic immune responses, contributing to unresponsiveness to self constituents and non-self antigens. The development and function of Treg cells depend on the expression of the transcription factor fork-head box P3 (FoxP3) [1]. Treg cells are also influenced by cytokines, including IL-2, IL-10 and TGF-β [2], and can suppress immunity through cell-to-cell contact-dependent suppression, cytokine control, and killing of effector cells [3]–[6]. However, the mechanisms of suppression are not known well.

It is necessary to control the magnitude of Treg-mediated suppression for the benefit of the host because too much suppression will result in more immune suppression and render the host susceptible to infection and cancer [2]. Elevated proportions of Tregs among tumor-infiltrating lymphocytes have been described in many types of cancer, including ovarian carcinoma [7]. In addition, results of several studies have shown that increased Treg infiltration in ovarian cancer is associated with poor survival [8]–[10].

Conventional modalities for malignancies are surgery, chemotherapy, or radiation therapy. Currently, the standard treatment of ovarian carcinoma is surgical intervention followed by platinum-containing chemotherapy [11], [12]. Because of the lack of symptoms and adequate screening methods in the early stage, about 75% of cancer patients are diagnosed as advanced diseases [13] and their five-year overall survival rate is only 20–30% [14]. To achieve better ovarian cancer management, many modalities are still being explored. In the viewpoint of immunology, immune manipulation may be an attractive alternative approach because it has the specificity to discriminate between neoplastic and non-neoplastic cells [15]. Although the precise mechanisms of host immune responses to tumor cells are still unclear, malignant tumors have been immunogenic in some cancer sites, including ovarian carcinoma [8], [16], [17]. Therefore, clinical trials of using immunologic modalities for ovarian cancer patients have been ongoing over the last two decades [18], but the most effective immune manipulation for ovarian carcinoma is still eagerly awaited.

Literature about detailed changes of host effective and suppressive immunities, including immunocytes and cytokine profiles during tumor progression, is rare. Thus, this study used the ascitogenic animal model with WF-3 tumor cell line [19], which can generate tumor sharing similar to the morphologic features of ovarian tumors, to verify the immunologic findings of ascites of ovarian cancer patients. A series of experiments were also designed to explore the dynamically systemic and local immune responses of hosts during tumor progression using this model, while *in vivo* Treg cells depletion experiments were performed to demonstrate whether the depletion of Tregs could reverse the imbalance between pro- and anti-tumor immunities during tumor progression and if the antigen-specific immunity could be generated. Lastly, through the survival analysis of mice treated with Abs to Tregs, the proposed modality of immune manipulation was investigated in the study.

## Materials and Methods

### Patients and specimens

Twenty patients (10 early stage diseases and 10 advanced diseases) with ovarian carcinoma undergoing staging or debulking surgery were recruited. Stages I and II diseases were defined as early stages while stages III and IV were defined as advanced stages. The Institutional Review Board reviewed and approved the study protocol. The collection of cancerous tissue, ascites, and peripheral PBMCs were acquired after the patients signed informed consent. These ascites specimens were separated into supernatant and cellular components by centrifugation at 2000 rpm for 5 minutes. The supernatant was stored at −20°C and the cells were stored at −135°C until analysis. The disease stage of the ovarian cancer patients was based on the criteria of the International Federation of Gynecology and Obstetrics (FIGO) [20].

### Tumor cell line

The generation of WF-3 tumor cells was as previously described and maintained in RPMI-1640, supplemented with 10% (volume/volume) fetal bovine serum, 50 U/mL penicillin/streptomycin, 2 mM L-glutamine, 1 mM sodium pyruvate, 2 mM non-essential amino acids, and 0.4 mg/mL G418 at 37°C with 5% carbon dioxide [19].

### Mice

Six-to-eight week-old female C57BL/6J mice were bred in and purchased from the animal facility of the National Taiwan University Hospital (Taipei, Taiwan). All animal procedures were conducted according to approved protocols and in accordance with recommendations for the proper use and care of laboratory animals.

### Collection of ascites and tumor-associated cells (TACs)

The WF-3 tumor cells (5×10^4^/mouse) were injected intra-peritoneally (6 mice per group) and the mice were sacrificed on days 14 and 49 post-injection. One ml phosphate-buffered saline (PBS) was injected into the peritoneal cavity and intra-peritoneal fluid was collected on day 14 after tumor injection while ascites was collected directly from mice 49 days after tumor injection. The ascites were separated into supernatant and cellular components as described earlier. The supernatant was stored at −20°C whereas cells defined as tumor-associated cells were stored at −135°C until analysis.

### Surface marker staining and flow cytometry of splenocytes and TACs

For the animal part, the mice were first injected with WF-3 and sacrificed after tumor challenge as described earlier. The splenocytes were treated and obtained as described previously [21]. The splenocytes were then used directly or stored at −135°C until further experiments.

The mice splenocytes and TACs were stained with fluorescein isothiocyanate (FITC)-conjugated anti-mouse CD3 (Biolegend, San Diego, CA), phycoerythrin (PE)-conjugated anti-mouse CD4 (Biolegend), PE-conjugated anti-mouse CD8 (Biolegend), PE/Cy5-conjugated anti-mouse CD4 (eBioscience, San Diego, CA), PE-conjugated anti-mouse CD25 (eBioscience), PE-conjugated anti-mouse CD19 (eBioscience), PE-conjugated anti-mouse NK1.1(Biolegend), or PE-conjugated anti-mouse CD223 (eBioscience) for different experiments [22]. Flow cytometry assays and analyses were performed using a Becton Dickinson FACScan (Becton Dickinson, Franklin Lakes, NJ) with CELLQuest software.

For the human part, the human TACs were stained with FITC-conjugated anti-human CD3 (Biolegend), FITC-conjugated anti-human Lin (Biolegend), PE-Cy5-conjugated anti-human CD4 (Biolegend), PE-Cy5-conjugated anti-human CD33 (Biolegend), PE-conjugated anti-human CD11b (Biolegend), PE-conjugated anti-human CD8 (BD Biosciences, San Diego, CA), or PE-conjugated anti-human CD25 (Biolegend) in different experiments, and analyzed by flow cytometry as described earlier.

### Immuno-histochemistry for CD4^+^FoxP3^+^ Treg cells

Immuno-histochemistry studies of Treg cells in murine spleens were performed with some modifications [23]. Briefly, eight-micrometer cryostat sections were obtained from unfixed tissue embedded in optimal cutting temperature (OCT) compound. After fixation with cold methanol (−20°C) for 20 min, the sections were incubated with 5% fetal bovine serum (FBS) for 10 min. Subsequently, the sections were incubated at 4°C overnight with the primary antibody, including rat anti-mouse CD4 antibody (Abcam, Cambridge, MA) and rabbit anti-mouse FoxP3 antibody (Abcam), and then washed three times in PBS for 15 min.

After incubation with the primary antibody, the sections were then incubated at room temperature for 1–2 hours with appropriate secondary antibodies like anti-rat secondary antibody-FITC (Abcam) and anti-rabbit secondary antibody-H&L-F(ab)2 fragment (Abcam) in PBS containing 0.5% FBS, followed by counter-staining by Hoechst33342 (Sigma-Aldrich, St. Louis, MO). After several washings with PBS, the sections were cover-slipped using anti-fade mounting medium (Invitrogen, Carlsbad, CA) and analyzed by confocal microscopy (Leica TCS SP2, Heidelberg, Germany).

### Characterization of Tregs by flow cytometry

To identify the Treg cells in murine splenocytes and TACs, splenocytes and TACs were first stained with PE/Cy5-conjugated anti-mouse CD4 (eBioscience) and PE-conjugated anti-mouse CD25 (eBioscience) for cell surface markers. Fluorescein isothiocyanate-conjugated anti-mouse FoxP3 (eBioscience) for intracellular staining were performed as described previously [24]. Staining was characterized by flow cytometric analysis as described earlier.

For the human experiments, the TACs of the ascites were stained with PE/Cy5-conjugated anti-human CD4 (Biolegend) and PE-conjugated anti-human CD25 (Biolegend) for cell surface markers, and Alexa Fluor® 488 anti-human FoxP3 (Biolegend) for intracellular staining to identify human Treg cells in ascites. Staining was analyzed by flow cytometry as described earlier.

### Extraction of RNA in murine splenocytes and various cytokine expressions by reverse-transcription polymerase chain reaction (RT-PCR)

Total RNA of murine splenocytes was first isolated by TRIzol reagent following the manufacturer's instructions (Invitrogen). To detect the RNA expression of various cytokines in the splenocytes, RT-PCR with primers specific for interleukin (IL)-4, 6, 10, and 12, tumor necrosis factor-alpha (TNF-α), interferon-gamma (IFN-γ) and GAPDH were done. The sequences of PCR primers were listed as follows: mouse IL-4 (forward primer: 5′-TCAACCCCCAGCTAGTTGTC-3′; reverse primer: 5′-ATCGAAAAGCCCGAAAGAGT-3′), mouse IL-6 (forward primer: 5′-GTTCTCTGGGAAATCGTGGA-3′; reverse primer: 5′-GGAAATTGGGGTAGGAAGGA-3′), mouse IL-10 (forward primer: 5′-TGCTATGCTGCCTGCTCTTA-3′; reverse primer: 5′-TTTTCACAGGGGAGAAATCG-3′), mouse IL-12 (forward primer: 5′-CACGCCTGAAGAAGATGACA-3′; reverse primer: 5′-AGTCCCTTTGGTCCAGTGTG-3′), mouse TNF-α (forward primer: 5′-ACGGCATGGATCTCAAAGAC-3′; reverse primer: 5′-CGGACTCCGCAAAGTCTAAG-3′), and mouse IFN-γ (forward primer: 5′-GCTTTGCAGCTCTTCCTCAT-3′; reverse primer: 5′-TGAGCTCATTGAATGCTTGG-3′). The GAPDH forward primer was 5′-ACCCAGAAGACTGTGGATGG-3′, and the reverse primer was 5′-TGCTGTAGCCAAATTCGTTG-3′. The amplification products were separated by 1% agarose gel electrophoresis and visualized after staining with ethidium bromide.

### Enzyme-linked immunosorbent assays (ELISA) of cytokines in ascites of WF-3 tumor-bearing mice and ovarian cancer patients

Direct ELISAs of human IL-4, 5, 6, 9, 10, 12,13, 17, TNF-α, IFN-γ, and transforming growth factor-beta (TGF-β) (e-Bioscience) and murine IL-4, 6, 10, 12, TNF-α, IFN-γ, and TGF-β (e-Bioscience) in ascites were performed based on the manufacturer's instructions [25].

### IFN-γ ELISPOT assays

The ELISPOT assays of mesothelin-specific CD8^+^ T cells in murine splenocytes were performed with some modifications [26]. The 96-well filtration plates (Millipore, Bedford, MA) were coated with 5 µg/ml anti-mouse INF-γ antibody (BD Biosciences) in 100 µl PBS. After being incubated overnight at 4°C, the wells were washed and blocked with culture medium containing 10% FBS. The mice of various groups were treated and the splenocytes were collected as described earlier.

Different groups of splenocytes were serially cultured with 10 µg/ml mesothelin peptide (aa 406–414) for 48 h at 37°C, 5% CO_2_. Following culture, the plate was washed and then incubated with 2 µg/ml biotinylated anti-mouse IFN-antibody (BD Biosciences) in 100 µl PBS at 4°C overnight. After subsequent washing, 1.2 µg/ml avidin-alkaline phosphatase (Sigma-Aldrich) in 100 µl PBS was added and the plates were incubated for two hours at room temperature. Afterwards, spots were developed by adding 100 µl 5-bromo-4-chloro-3-indolyl phosphate/nitroblue tetrazolium solution (Boehringer Mannheim, Indianapolis, IN) and incubation at room temperature for 20 min. The reactions were stopped by discarding the substrate and washing the plates under tap water. The plates were then air-dried and the colored spots were counted using a dissecting microscope.

### In vivo antibody depletion experiments

The mAb PC61 (Bio X cell, West Lebanon, NH) was used for *in vivo* CD25 depletion [27]. Flow cytometric analysis revealed that 95% of the appropriate lymphocyte subsets were depleted, while other lymphocyte subsets remained within normal levels. Briefly, C57BL/6J mice (5 mice per group) were challenged with WF-3 tumor cells intra-peritoneally on day 0. Mice were sacrificed on days 14 or 49 for the immunologic profiling assays and the remaining mice (5 in each group) were kept until they died to obtain overall survival analysis. The total weight of tumors of each mouse was also measured when sacrificed. Depletion was terminated on the day of euthanasia. The splenocytes and ascites were harvested for immunocytes and cytokine analyses as described earlier.

The mAb 1D11.17.8 and mAb JES5-2A5 (Bio X cell) were used for *in vivo* neutralizing the effects of TGF-β and IL-10, respectively [28], [29]. In this experiment, the mice (5 mice per group) were challenged with WF-3 tumor cells intra-peritoneally on day 0. It was terminated on day 100. The overall survivals of mice would be analyzed.

### Statistical analysis

All of the data were expressed as mean±S.E. (standard error), which represented at least two different experiments. Data for the kinetic changes of immune effectors, regulatory T lymphocytes, cytokine expression, and total tumor weights were evaluated by one-way analysis of variance (ANOVA). The event time distributions for different mice in the survival experiments were compared using Kaplan-Meier method and log rank analysis. A *p*<0.05 was considered statistically significant.

## Results

### Immune profiles of immunocytes and cytokines in ascites changed between early- and advanced-stage ovarian cancer patients

**Figure 1:**
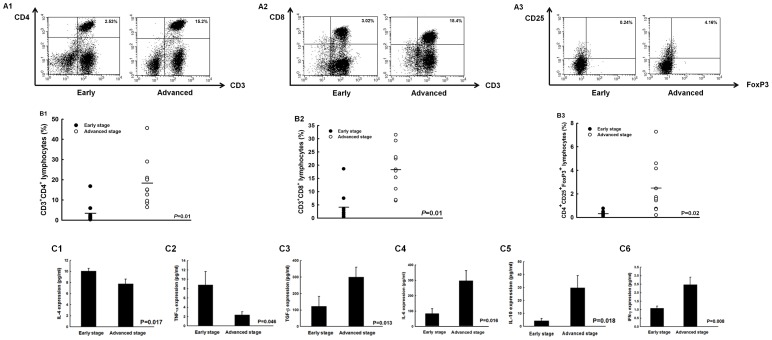
Different expressions of immune components in ascites of early- and advanced-stage ovarian cancer patients. (**A**) Representative figures of flow cytometric analyses of CD4^+^ helper T cells, CD8^+^ cytotoxic T lymphocytes, and CD4^+^CD25^+^ regulatory T lymphocytes (Treg cells) in TAL. A1, CD4^+^ helper T cells; A2, CD8^+^ cytotoxic T lymphocytes; A3, CD4^+^CD25^+^ Treg cells. (**B**) Percentages of CD4^+^ helper T lymphocytes, CD8^+^ cytotoxic T lymphocytes, and CD4^+^CD25^+^ regulatory T lymphocytes (Treg cells) in TAL. B1, CD4^+^ helper T cells; B2, CD8^+^ cytotoxic T lymphocytes; B3, CD4^+^CD25^+^ Treg cells. *Note*: The percentages of CD4^+^ T cells were significantly higher in patients with advanced stage ovarian cancer. (**C**) Concentrations of various cytokines in ascites of ovarian cancer patients. C1, IL-4; C2, TNF-α; C3, TGF-β; C4, IL-6; C5, IL-10; C6, IFN-γ. *Note*: The IL-4 and TNF-α concentrations decreased while the TGF-β, IL-6, IL-10, and IFN-γ concentrations increased from early to advanced stage.

The ascites of ovarian cancer patients was analyzed to evaluate the immune components of tumor micro-environment. Representative figures of flow cytometric analysis for various immunocytes, such as CD4^+^ helper, CD8^+^ cytotoxic, and regulatory T lymphocytes in ascites were shown in Figure 1A. The percentages of CD4^+^ helper T cells significantly increased in advanced-staged patients compared to those in patients with early stages (advanced 18.3±3.7% vs. early 3.4±1.6%, *p* = 0.01, one-way ANOVA) (Fig. 1B1). Similar phenomena were observed in the percentages of CD8^+^ cytotoxic T lymphocytes (advanced 18.3±2.7% vs. early 4.1±1.7%, *p* = 0.01, one-way ANOVA) (Fig. 1B2) and Treg cells (advanced 2.5±0.7% vs. early 0.3±0.1%, *p* = 0.02, one-way ANOVA) (Fig. 1B3). Moreover, the ratios of CD8^+^ T cell/Treg were significantly lower in patients with advanced stages than in patients with early stages (advanced 7.37±0.64 vs. early 14.25±3.11, *p* = 0.037, one-way ANOVA). However, the percentages of myeloid suppressor cells (MDSCs) in TACs between early- and advanced-stage ovarian cancer patients were not significantly different (*p* = 0.46, one-way ANOVA, Fig. S1A).

The IL-4 concentrations (10.1±0.5 pg/ml in early stages vs. 7.8±0.8 pg/ml in advanced stages, *p* = 0.017, one-way ANOVA) (Fig. 1C1) and TNF-α in ascites (8.8±2.8 pg/ml in early stages vs. 2.4±0.7 pg/ml in advanced stages, *p* = 0.046, one-way ANOVA) (Fig. 1C2) were significantly higher in patients with early-stage than in patients with advanced stages. In contrast, the other cytokines, including TGF-β (122.9±60.4 pg/ml in early stage vs. 300.9±58.5 pg/ml in advanced stage, *p* = 0.013, one-way ANOVA) (Fig. 1C3), IL-6 (83.7±32.0 pg/ml in early stage vs. 287.3±65.3 pg/ml in advanced stage, *p* = 0.016, one-way ANOVA) (Fig. 1C4), IL-10 (4.3±1.8 pg/ml in early stage vs. 29.9±9.3 pg/ml in advanced stage, *p* = 0.018, one-way ANOVA) (Fig. 1C5), and INF-γ (1.1±0.1 pg/ml in early stage vs. 2.5±0.4 pg/ml in advanced stage, *p* = 0.008, one-way ANOVA) (Fig. 1C6) in ascites were higher in patients with advanced stages than in patients with early stages. There were no differences in IL-5 (*p* = 0.26, one-way ANOVA), IL-9 (*p* = 0.23, one-way ANOVA), IL-12 (*p* = 0.54, one-way ANOVA), IL-13 (*p* = 0.43, one-way ANOVA), and IL-17 (*p* = 0.06, one-way ANOVA) expression levels between these two groups (Fig. S1B).

In this evaluation of ovarian cancer-related ascites, the expressions of host immune components were not constant between early- and advanced-stage ovarian cancer patients. The percentages of anti-tumor effectors were decreased but the percentage of immune suppressor, Treg was decreased as the disease progressed in this analysis. The expression levels of various cytokines were also different between early- and advanced-stage diseases.

### Percentages of systemic immune effector cells in splenocytes decreased with tumor progression

**Figure 2:**
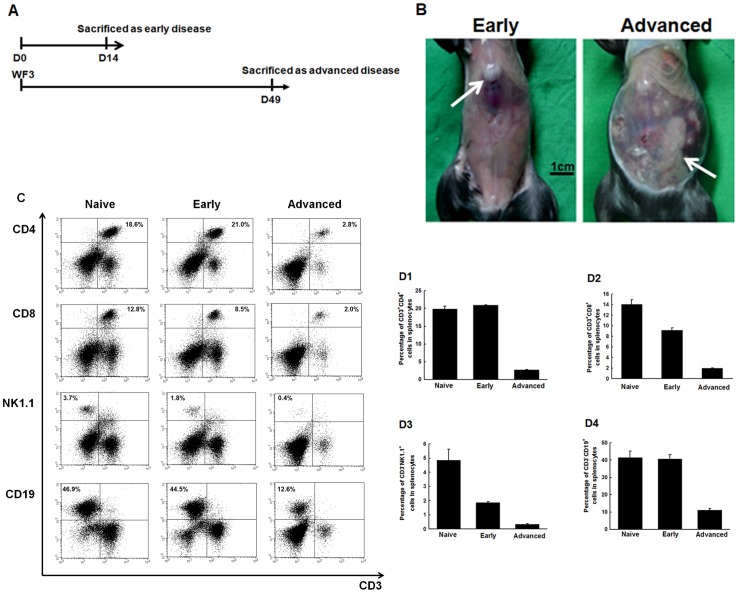
Alterations of systemic immune effector cells in splenocytes of mice challenged with PBS or WF-3 tumor cells. (**A**) Diagrammatic representation of the collection of specimens in early and advanced diseases. (**B**) Representative figures of mice after WF-3 challenge at days 14 and 49. *Note*: Only small tumors (arrow) with little ascites were identified in mice of early disease. However, disseminated tumor implants (arrows) with bloody ascites within the whole peritoneal cavity were noted in mice with advanced disease. (**C**) Representative figures of flow cytometric analyses of various kinds of lymphocytes in splenocytes. (**D**) Percentages of various kinds of lymphocytes in splenocytes of naïve mice and in mice of early and advanced diseases. D1, CD4^+^ helper T lymphocytes; D2, CD8^+^ cytotoxic T lymphocytes; D3, NK1.1^+^ natural killer cells; and D4, CD19^+^ B lymphocytes. *Note*: All of the percentages of systemic immune effector cells significantly decreased as the tumor progressed from early to advanced stage.

To further elucidate the dynamic changes of immunity profiles in human ovarian cancer, an ascitogenic animal model was used. The protocol of evaluating immunologic profiles of tumor-bearing mice in early or advanced diseases was shown in Figure 2A. The PBS-challenged mice were used as control (naïve group). Representative figures of tumor-bearing mice in the early and advanced disease stages were shown in Figure 2B. Only small tumors with little ascites were found in mice with early disease. However, disseminated tumor implants with bloody ascites were identified in mice with advanced disease.

Representative figures of flow cytometric analysis of immune effectors, such as CD4^+^ helper and CD8^+^ cytotoxic T cells, NK cells, and B lymphocytes in splenocytes, were shown in Figure 2C. The percentages of CD4^+^ helper T lymphocytes significantly decreased in mice with advanced disease (2.60±0.14%) compared to the naïve (19.81±0.80%) or early disease (20.89±0.16%) groups (*p*<0.001, one-way ANOVA) (Fig. 2D1). However, the percentages of CD8^+^ cytotoxic T lymphocytes decreased in the early disease (9.13±0.50%), but especially in the advanced disease (1.94±0.07%) compared to the naïve group (14.04±0.89%) (*p*<0.001, one-way ANOVA) (Fig. 2D2). The percentages of NK cells had similar phenomena as those of CD8^+^ cytotoxic T lymphocytes (naïve group 4.85±0.79%, early disease 1.87±0.07%, advanced disease 0.34±0.05%, *p*<0.001, one-way ANOVA) (Fig. 2D3). the B lymphocyte percentages in various groups were also similar to those of the CD4^+^ helper T lymphocytes (naïve group 41.40±3.69%, early disease 40.73±2.56%, advanced disease 11.06±1.05%, *p*<0.001, one-way ANOVA) (Fig. 2D4).

Between naïve group and early disease group,CD4^+^ helper T lymphocytes and B lymphocytes did not significantly decrease, but CD8^+^ cytotoxic T cells and NK cells did. However, all the systemic immune effector cells significantly decreased as compared to the naïve group when the tumor progressed to advanced stage in our results.

### Percentages of local immune effector cells in TACs of ascites changed with tumor progression

**Figure 3:**
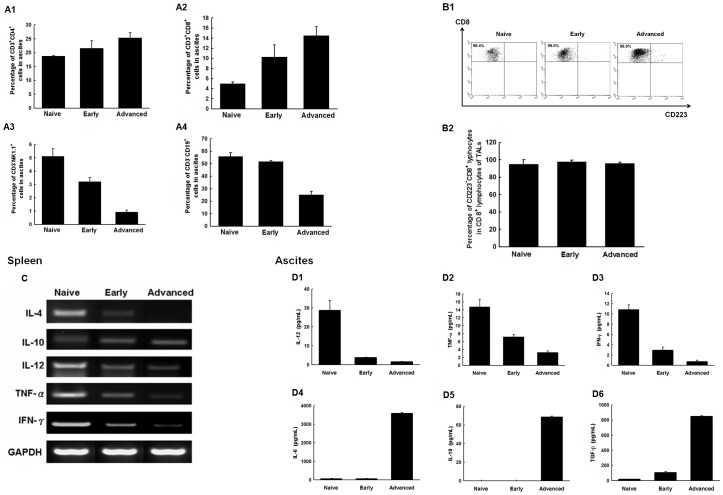
Kinetic changes of local immune effector cells in TACs of mice challenged with PBS or WF-3 tumor cells. (**A**) Percentages of various kinds of lymphocytes in TACs of naïve mice and mice of early and advanced diseases. A1, CD4^+^ helper T lymphocytes; A2, CD8^+^ cytotoxic T lymphocytes; A3, NK1.1^+^ natural killer cells; and A4, CD19^+^ B lymphocytes. *Note*: For helper and cytotoxic T lymphocytes, the percentages on TACs increased from early to advanced disease. However, the percentages of natural killer cells and lymphocytes in TACs significantly decreased with tumor progression. (**B**) CD223^−^CD8^+^ lymphocytes in TACs of mice. B1: Representative figures of flow cytometric analyses of CD223^−^CD8^+^ and CD223^+^CD8^+^ lymphocytes in TACs. **B2:** Percentages of CD223^−^CD8^+^ lymphocytes in TACs of mice. *Note*: Majority of CD8^+^ cytotoxic T lymphocytes were non-activated as the disease progressed to advanced status. (**C**) The RT-PCR of various cytokines in splenocytes of mice challenged with PBS or WF-3 tumor cells. *Note*: The expression levels of IL-4, IL-12, TNF-α, and INF-γ decreased but the expression levels of IL-10 increased gradually as the disease progressed. (**D**) The concentrations of various cytokines by ELISA in ascites of mice challenged with PBS or WF-3 tumor cells. D1, IL-12; D2, TNF-α; D3, IFN-γ; D4, IL-6; D5, IL-10; D6, TGF-β. *Note*: The concentrations of IL-6, IL-10, and TGF-β were elevated but those of IL-12, TNF-α, and IFN-γ decreased as the tumor progressed.

The change of effector immunocytes in the local tumor environment was then evaluated by flow cytometric analysis. Representative figures of flow cytometric analysis of various immune effector cells were shown in Figure S2. The percentages of CD4^+^ helper T lymphocytes increased as the disease progressed to the advanced stage (naïve 18.6±0.24%, early 21.6±2.65%, advanced 25.4±1.79%, *p* = 0.002, one-way ANOVA) (Fig. 3A1). The percentages of CD8^+^ cytotoxic T cells in TACs also increased with disease progression (naïve 5.01±0.26%, early 10.3±2.41%, advanced 14.5±1.81%, *p*<0.001, one-way ANOVA) (Fig. 3A2).

In contrast, the percentages of NK cells (naïve 5.12±0.56%, early 3.20±0.32%, advanced 0.93±0.14%, *p*<0.001, one-way ANOVA) and B lymphocytes (naïve 55.90±3.03%, early 51.6±1.01%, advanced 25.10±2.69%, *p*<0.001, one-way ANOVA) in TACs decreased with disease progression (Figs. 3A3 and 3A4).

The number of T lymphocytes increased in the local tumor environment as the disease progressed, but the numbers of NK cells and B lymphocytes decreased. Moreover, in this intra-peritoneal tumor model, the changes of immune effector cells were different between the systemic (spleen) and the local (ascites) tumor environment as the disease progressed.

### The numbers of activated CD8^+^ cytotoxic T lymphocytes in the TACs of ascites did not increase between early and advanced diseases

To further evaluate whether the higher number of CD8^+^ cytotoxic T lymphocytes in the TACs of advanced disease were activated or not, the surface marker expression of CD223, which was the activated marker of T lymphocytes, was detected [30]. Representative figures of activated and non-activated cytotoxic CD8^+^ T lymphocytes were shown in Figure 3B1. The percentages of non-activated CD8^+^ cytotoxic T lymphocytes in the TACs of the naïve group (94.8±5.2%), in the early disease group (97.62±1.80%), and in the advanced disease group (95.80±1.63%) had no statistical difference (*p* = 0.68, one-way ANOVA) (Fig. 3B2). Therefore, majority of the CD8^+^ cytotoxic T lymphocytes were not activated in the local tumor micro-environment, although the numbers of CD8^+^ T lymphocytes increased as the disease progressed.

### Dynamic changes of cytokine profiles in systemic immunity and local tumor environment with various disease severities

The RNA expression levels of various cytokines were evaluated in the splenocytes of mice by RT-PCR as systemic immunity. The RNA expression levels of IL-4, IL-12, TNF-α, and INF-γ decreased as the disease progressed. In contrast, the RNA expression levels of anti-inflammatory cytokines like IL-10 increased (Fig. 3C).

The concentrations of cytokine profiles in ascites as local immunity were further evaluated by ELISA. The concentrations of cytokines like IL-12 (naïve 28.7±5.4, early 3.7±0.2, advanced 1.5±0.2 pg/ml, *p* = 0.001, one-way ANOVA), TNF-α (naïve 14.7±1.9, early 7.2±0.6, advanced 3.2±0.5 pg/ml, *p* = 0.001, one-way ANOVA), and IFN-γ (naïve 10.9±0.9, early 2.9±0.6, advanced 0.7±0.3 pg/ml, *p*<0.001, one-way ANOVA) decreased gradually as the tumor burden increased (Figs. 3D1–3D3). However, the concentrations of other cytokines, including IL-6 (naïve 70.8±0.5, early 70.7±0.8, advanced 3585.6±53.4 pg/ml, *p*<0.001, one-way ANOVA), IL-10 (naïve 0.0±0.0, early 0.0±0.0, advanced 68.8±0.7 pg/ml, *p*<0.001, one-way ANOVA), and TGF-β (naïve 17.9±1.5, early 107.5±13.6, advanced 848.9±9.0 pg/ml, *p*<0.001, one-way ANOVA) were significantly elevated when the tumor progressed (Figs. 3D4–3D6).

### The percentages of Tregs in systemic or local tumor environments increased with disease progression

**Figure 4:**
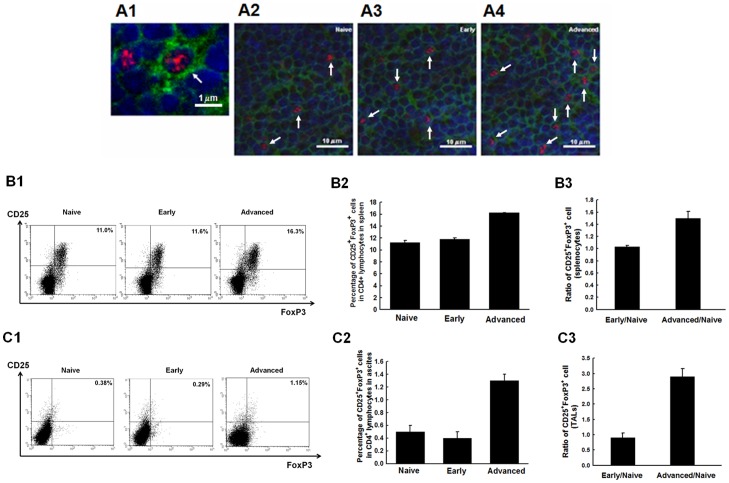
Kinetic changes of Treg cells in the splenocytes and TACs of mice challenged with PBS or WF-3 tumor cells. (**A**) Representative figures of CD4^+^FoxP3^+^ Treg cells in the spleen of mice by immuno-histochemistry staining observed under confocal microscopy. A1, A single CD4^+^FoxP3^+^ regulatory T cell (blue: nucleus, green: CD4, red: FoxP3); A2, PBS group; A3, WF-3 group with early disease; A4, WF-3 group with advanced disease (arrows: CD4^+^FoxP3^+^ Treg cells). (**B**) CD4^+^CD25^+^FoxP3^+^ Treg cells in splenocytes. B1: Representative figures of flow cytometric analyses. B2: The bar figures of CD4^+^CD25^+^FoxP3^+^ Treg cells in various groups. B3: The ratios of CD4^+^CD25^+^FoxP3^+^ Treg cells. *Note:* Referenced with the naïve group, Treg cells of the advanced stage group were 1.5-fold higher in splenocytes. (**C**) CD4^+^CD25^+^FoxP3^+^ Treg cells in TACs. C1: Representative figures of flow cytometric analysese. C2: The bar figures of CD4^+^CD25^+^FoxP3^+^ Treg cells in various groups. C3: The bar figures of the ratios of CD4^+^CD25^+^FoxP3^+^ Treg cells in various groups. *Note:* Referenced with the naïve group, Treg cells were 2.9-fold higher in TACs of the advanced stage group.

The changes in immuno-suppressor cells such as regulatory T cells (Tregs) in the systemic and local tumor environments of various disease severities were then evaluated. The representative figures of a single CD4^+^FoxP3^+^ Treg cell and Treg cells of the spleen in various groups like naïve mice, mice with early disease, and mice with advanced disease by immuno-histochemistry or immuno-fluorescence staining were shown in Figure 4A. Representative figures of Treg cells in splenocytes (Fig. 4B1) and TACs (Fig. 4C1) by flow cytometry were also shown. The percentages of Treg cells in the splenocytes (naïve 11.28±0.33%, early 11.80±0.22%, advanced 16.24±0.02%, *p*<0.001, one-way ANOVA) (Fig. 4B2) and in TACs (naïve 0.51±0.11%, early 0.42±0.10%, advanced 1.32±0.09%, *p*<0.001, one-way ANOVA) (Fig. 4C2) were significantly higher in the advanced group than in the naïve and early disease groups. However, the percentages of Treg cells were not different between the naïve and early disease in either the splenocytes or the TACs.

The ratios of Treg cells in splenocytes and TACs in the early and advanced diseases were further evaluated with the percentages of Treg cells in the naïve group as baseline. The Treg cells were 1.5-fold higher in splenocytes (Fig. 4B3) and 2.9-fold higher in TACs (Fig. 4C3) in the advanced disease. The ratios of CD8^+^ cytotoxic T cell/Treg cell were further calculated. The murine CD8^+^ T cell/Treg in splenocytes (advanced 0.11±0.01 vs. early 1.39±0.04, p = 0.001) and in TACs (advanced 12.49±0.18 vs. early 34.79±4.54, p = 0.039) were lower in the advanced disease than in the early disease.

The results indicated that regulatory T lymphocytes significantly increased in the advanced disease, but not in the early disease, in both the local and systemic environments.

### Depletion of Tregs with kinetic low-dose CD25 Ab generated more potent *in vivo* anti-tumor effects

**Figure 5:**
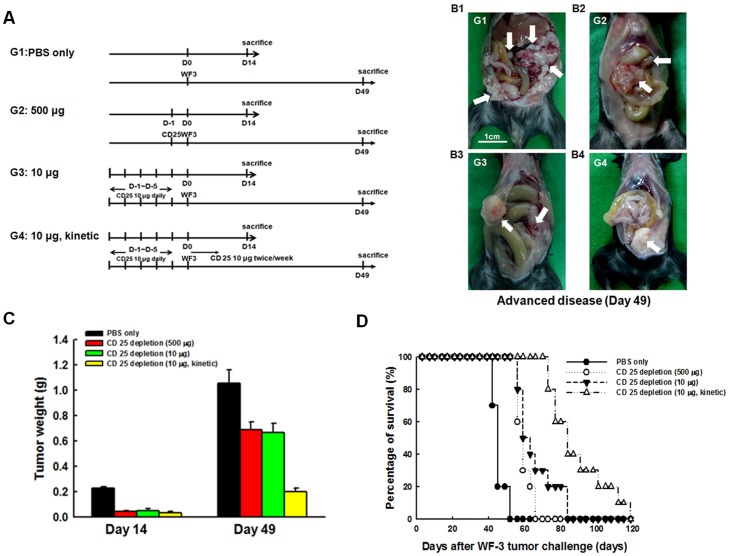
Tumor growth of WF-3-challenged mice treated with PBS or CD25 monoclonal antibody. (**A**) Diagrammatic representation of the different treatment protocols of CD25 monoclonal antibody. (**B**) Representative figures of mice treated with PBS and various protocols of CD25 monoclonal antibody. B1, PBS only; B2 (sequential high dose CD25 Ab group), 500 µg of CD25 monoclonal antibody for 1 day before WF-3 tumor challenge; B3 (sequential low dose CD25 Ab group), 10 µg of CD25 monoclonal antibody for 5 days before WF-3 tumor challenge; B4 (kinetic low dose CD25 Ab group), 10 µg of CD25 monoclonal antibody for 5 days before WF-3 tumor challenge and 10 µg of CD25 monoclonal antibody twice per week continuously after WF-3 tumor challenge (arrows: WF-3 tumor masses). (**C**) Intra-peritoneal tumor weights of mice treated with PBS or CD25 monoclonal antibody. *Note*: The total tumor weights of various CD25 Ab depletion groups were lower than those of PBS-treated group mice on day 14 after WF-3 tumor challenge. However, the intra-peritoneal tumor weights of the kinetic low dose CD25 Ab group were lowest compared to the other groups on day 49 after tumor challenge. (**D**) Survival curves of mice treated with PBS or CD25 monoclonal antibody. *Note*: The *in vivo* CD25 antibody depletion regardless of groups extended survival of the mice compared to those treated with PBS only. However, the kinetic low-dose CD25 Ab group had the longest survival time after WF-3 tumor challenge.

To explore whether the depletion of Tregs played an important role in delaying tumor progression and improving the survival of the tumor-bearing mice, the mAb PC61 was used for *in vivo* CD25 depletion via protocols of *in vivo* Treg cell depletion (Fig. 5A). Representative figures of tumor-bearing mice in various *in vivo* Treg cell depletion groups were shown in Fig. 5B. The intra-peritoneal tumor weights of the mice were significantly lower in the CD25 Ab depletion groups compared to those in the PBS group on the 14^th^ day after tumor challenge (PBS group 0.230±0.010 g, sequential high dose CD25 Ab group 0.045±0.005 g, sequential low dose CD25 Ab group 0.053±0.013 g, kinetic low dose CD25 Ab group 0.037±0.001 g, *p*<0.001, one-way ANOVA) (Fig. 5C). However, the tumor weights were not different among the three CD25 Ab depletion groups (*p* = 0.76, one-way ANOVA).

On the 49^th^ day after tumor challenge, the kinetic low-dose CD25 Ab depletion group had significantly lower intra-peritoneal tumor weights (0.20±0.03 g) than the sequential high-dose (0.69±0.06 g) and sequential low-dose (0.67±0.07 g) CD25 Ab deletion groups (*p* = 0.001, one-way ANOVA) (Fig. 5C). Mice challenged with WF-3 tumor cells, when treated with kinetic low dose CD25 Ab, also had significantly longer survival than the other three groups (*p*<0.001, log rank test) (Fig. 5D). Therefore, mice treated with kinetic low-dose CD 25 Ab could have less tumor amounts and prolonged survival time.

### Depletion of Tregs with *in vivo* kinetic low-dose CD25 Ab enhanced the anti-tumor immunologic profiles

**Figure 6:**
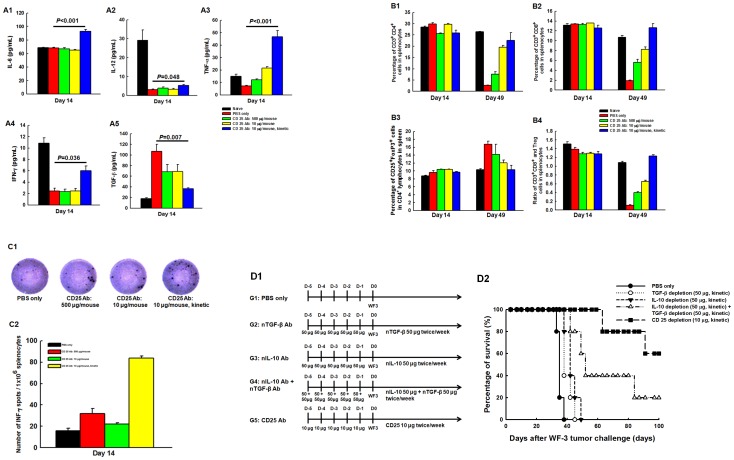
The concentrations of cytokine profiles in ascites and kinetic changes of various lymphocytes in splenocytes of WF-3-challenged mice treated with PBS or CD25 monoclonal antibody. (**A**) Concentrations of various cytokines in ascites. A1, IL-6; A2, IL-12; A3, TNF-α; A4, IFN-γ; A5, TGF-β. *Note*: Compared to mice treated with PBS only, the concentrations of cytokines like IL-6, IL-12, TNF-α, and IFN-γ were significantly elevated in mice treated with kinetic low-dose CD25 antibody than those in the other groups. The concentrations of TGF-β significantly decreased in the kinetic low-dose CD25 Ab group compared to the other groups. (**B**) Percentages of various lymphocytes. B1, CD3^+^CD4^+^ help T lymphocytes. *Note*: The percentages of CD4^+^ T lymphocytes in the splenocytes were not different between the PBS and CD25 Ab depletion groups on day 14 after WF-3 tumor challenge. However, the kinetic low-dose CD25 Ab depletion group had higher percentages of CD4^+^ helper T cells than the PBS and the other two sequential CD25 Ab depletion groups after 49 days of tumor challenge. B2, CD3^+^CD8^+^ cytotoxic T lymphocytes. *Note*: The percentages of CD8^+^ T cells were not different between the PBS and CD25 Ab depletion groups on 14 days after tumor challenge. The kinetic low-dose CD25 Ab depletion group had higher percentages of CD8^+^ cytotoxic T cells than the other groups on day 49 after tumor challenge. B3, CD4^+^CD25^+^Foxp3^+^ Treg cells. *Note*: The percentages of Treg cells in the splenocytes were not different among these groups on day 14 after tumor challenge. However, the kinetic low-dose CD25 Ab depletion group had lower percentages of Treg cells than the other groups on day 49. B4, ratios of cytotoxic T lymphocytes/Treg cells. *Note*: The ratios of CD8^+^ cytotoxic T cells/Treg cells in the kinetic low-dose CD25 Ab depletion group were highest among the four groups. (**C**) Mesothelin-specific IFN-γ ELISPOT assays of splenocytes in the PBS and CD25 Ab-treated groups. C1, the representative figures of mesothelin-specific IFN-γ ELISPOT assays of splenocytes; C2, bar figures of the numbers of IFN-γ-secreting, mesothelin-specific T lymphocytes in various groups. *Note:* The kinetic low-dose CD25 Ab depletion group generated the highest numbers of IFN-γ-secreting, mesothelin-specific T lymphocytes compared to the other groups. (**D**) Survivals of mice treated with PBS, neutralizing TGF-β, neutralizing IL-10 or CD25 monoclonal antibodies. D1, Diagrammatic representation of the different treatment protocols of neutralizing TGF-β, neutralizing IL-10 or CD25 monoclonal antibody; D2, Survival curves of mice treated with PBS, neutralizing TGF-β, neutralizing IL-10, neutralizing TGF-β and neutralizing IL-10 or CD25 monoclonal antibody. *Note:* The mice treated with kinetic low-dose CD25 Ab had the longest survival time after WF-3 tumor challenge when compared to the other mice treated with PBS, neutralizing TGF-β, neutralizing IL-10 or neutralizing TGF-β and neutralizing IL-10 monoclonal antibodies.

To elucidate the influence of host immunity in WF-3-challenged mice undergoing kinetic low-dose CD25 Ab, immunologic profiles were evaluated. Mice treated with kinetic low-dose CD25 Ab had significantly higher concentrations of IL-6 (*p*<0.001, one-way ANOVA) (Fig. 6A1), IL-12 (*p* = 0.048, one-way ANOVA) (Fig. 6A2), TNF-α (*p*<0.001, one-way ANOVA) (Fig. 6A3), and INF-γ (*p* = 0.036, one-way ANOVA) (Fig. 6A4), but lower concentrations of TGF-β (*p* = 0.007, one-way ANOVA) (Fig. 6A5) in ascites compared to the other groups.

The kinetic low-dose CD25 Ab depletion group also had significantly higher percentages of CD4^+^ helper (*p* = 0.001, one-way ANOVA) (Fig. 6B1) and CD8^+^ cytotoxic T lymphocytes (*p*<0.001, one-way ANOVA) (Fig. 6B2) in splenocytes than in PBS and the other two sequential CD25 Ab depletion groups after 49 days of WF-3 tumor challenge. In contrast, the kinetic low-dose CD25 Ab depletion group had lower percentages of Treg cells compared to the other groups (*p* = 0.045, one-way ANOVA) (Fig. 6B3).

The ratios of CD8^+^ cytotoxic T cells/Treg cells of the TACs in the kinetic low-dose CD25 Ab depletion group were also highest among the naïve, PBS-treated, and the three CD25 Ab depletion groups (*p*<0.001, one-way ANOVA) (Fig. 6B4). However, the percentages of CD4^+^ helper T cells (*p* = 0.06, one-way ANOVA) (Fig. 6B1), CD8^+^ cytotoxic T cells (*p* = 0.27, one-way ANOVA) (Fig. 6B2), or Treg cells (*p* = 0.22, one-way ANOVA) (Fig. 6B3) in the splenocytes were not different between among these groups after the 14-day WF-3 tumor challenge.

In addition, this study evaluated if the *in vivo* CD25 Ab depletion could generate anti-tumor effects via enhancing antigen-specific immunity using mesothelin, a novel ovarian tumor-associated antigen. Representative figures of mesothelin-specific IFN-γ ELISPOT assays of splenocytes were shown in Figure 6C1. The kinetic low-dose CD25 Ab depletion group generated the highest numbers of IFN-γ-secreting, mesothelin-specific T lymphocytes (83.7±2.0) compared to the other groups after 14 days of WF-3 tumor challenge (PBS group 16.0±2.3, sequential high dose CD25 Ab group 32.0±4.6, sequential low dose CD25 Ab group 22.0±1.2, *p*<0.001, one-way ANOVA) (Fig. 6C2).

These above-mentioned results demonstrated that kinetically depleting the immuno-suppressive Treg cells could enhance effective host anti-tumor immunity. Therefore, we would like to explore the impacts of different treatment protocols with neutralizing TGF-β, neutralizing IL-10 or Treg depletion with CD25 monoclonal antibody on mice survival. The experimental schedules were shown in Figure 6D1. Our result showed that when mice were treated with monoclonal antibodies, their survival time was longer than that of mice treated with PBS (neutralizing TGF-β group, *p* = 0.03, log rank test; neutralizing IL-10 group, *p* = 0.002, log rank test; neutralizing TGF-β and neutralizing IL-10 group, *p*<0.001, log rank test; kinetic low-dose CD25 Ab group, *p*<0.001, log rank test) (Fig. 6D2). But, among the mice with monoclonal antibody treatment, those in the kinetic low-dose CD25 Ab group still had the longest survival time with significance when compared to the other mice in the neutralizing TGF-β group, neutralizing IL-10 group and neutralizing TGF-β and neutralizing IL-10 group (*p*<0.001, log rank test) (Fig. 6D2).

## Discussion

Immunogenicity is the ability of antigens to elicit an immune response. Host anti-tumor immunity can be induced because of the existence of tumor-specific and/or tumor-associated antigens. To date, over 1000 human tumor antigens have been established in the human cancer immunome database (http://ludwig-sun5.unil.ch/CancerImmunomeDB/). Ovarian carcinoma has been proven to be immunogenic in previous investigations [16], [17], [18]. The inflammation of lesion site has been associated, in part, with various regulatory components that can modify immune cell recruitment, phenotype, and function. These can result in the accumulation of naive and/or functionally erroneous T cell sub-populations at such sites [31], [32]. In the present analysis of human and murine ascites, the percentages of CD4^+^ and CD8^+^ T lymphocytes in the TACs of ascites are significantly increased in advanced disease compared to early stage disease (Figs. 1 and 3).

However, the homeostasis between effective and suppressive immunities, including cytokines and immunocytes, has an important impact on tumor progression. When diseases progress to advanced stages, the expressions of pro-inflammatory cytokines such as IL-4 and TNF-α are significantly suppressed [33], [34]. Other reports have also shown that increased anti-inflammatory cytokines like IL-10 and TGF-β can help Tregs suppress the function of antigen-presenting cells and arrest the activation of effector T lymphocytes during tumor progression [2], [35], [36]. In the present analysis of human ascites, pro-inflammatory cytokines are significantly lower and anti-inflammatory cytokines are significantly higher in advanced diseases than in early stage diseases (Fig. 1C). The imbalance between pro- and anti-inflammatory cytokines during tumor progression is shown to be immuno-suppressive in the animal model (Fig. 3). Nevertheless, alterations of INF-γ in human (Fig. 1C6) and murine (Fig. 3D3) in ascites are not identical, which can be explained by the fact that aside from immuno-surveillance, high levels of INF-γ in advanced diseases can generate tumor variants with reduced immunogenicity, which is related to the process of “cancer immuno-editing” [37]–[40].

IL-17 is involved in mediating inflammatory responses [41], [42]. Less amount of IL-17 can be detected in more advanced ovarian cancer-associated ascites (*p* = 0.03) [43]. However, the expression level of IL-17 in early-stage ovarian cancer is only marginally significantly (*p* = 0.06) higher than that in advanced disease (Fig. S1B5) in our analysis. This is because that the number of ovarian cancer patients in this study is small (only 20 cases). As more patients could be recruited, the expression level of IL-17 between early- and advanced-disease might be significant. The correlation between the level of IL-17 and clinical outcomes of ovarian cancer patients could also be elucidated.

T lymphocytes are a major component of cellular immune response and are the essential cells required for anti-tumor immunity [35]. For ovarian carcinoma, tumor specific CD8^+^ T cells can induce autologous tumor cell lysis *in vitro* [44]. However, the CD4^+^CD25^+^FoxP3^+^ Tregs suppress tumor-specific T-cell immunity and increased Tregs in the tumor micro-environment can be related to the disease severity and poor outcome of ovarian carcinoma [7], [8]. Sato et al. have also demonstrated that the survivals of ovarian cancer patients with high versus low CD8^+^/Treg ratios are 58 versus 23 months (*p* = 0.0002) [45]. In the present animal model, the increased percentages of Tregs are noted in systemic (splenocytes) and local (TACs) immunities during tumor progression (Fig. 4), but the alterations of CD8^+^ T cells are different (Figs. 2D2 and 3B2). When the ratios of murine CD8^+^ T cell/Treg in splenocytes and TACs are further analyzed, the ratios are lower in advanced disease than in early disease. Thus, alterations of ratios of CD8^+^ T cell/Treg can better illustrate disease severity rather than the changes of percentages of immunocytes. Moreover, even though the CD8^+^ T lymphocytes of TACs increase during tumor progression (Fig. 2D2), most of the CD8^+^ cytotoxic T lymphocytes are not activated in the present study (Figs. 3C and 3D).

The reduction of Tregs or the attenuation of their function can increase anti-tumor immunity and effects [8], [27], [46], [47]. In the present animal model, there are decreased total tumor volumes and weights, and longer survival in the mice with Treg cells depleted by CD25 monoclonal antibody than in those treated with PBS only (Fig. 5). Kinetic low-dose antibody depletion of Treg cell can generate anti-tumor immunity by enhancing the pro-inflammatory cytokines and the anti-tumor effector lymphocytes, including antigen-specific IFN-γ-secreting CD8^+^ T cells (Fig. 6A–6C). In addition, those mice treated with kinetic low-dose CD25 Ab would have the longest survival time when compared to the other mice treated with neutralizing TGF-β Ab, neutralizing IL-10 Ab and neutralizing TGF-β and neutralizing IL-10 Abs (Fig. 6D). The reason might be that Treg cells can suppress host immunity through cell-to-cell contact-dependent suppression, cytokine control, including TGF-β and IL-10 secretion and killing of effector cells [3]–[6], [48]–[50]. Therefore, more comprehensive immune components could be elicited by kinetic low-dose antibody depletion of Treg other than TGF-β, IL-10 or TGF-β and IL-10 neutralization.

For suppressing the function of Treg cells to enhance anti-tumor immunity, monoclonal antibodies specific to cell surface molecules that are predominantly expressed by Tregs or those specifically able to modulate Treg function have been developed. In addition to CD25 monoclonal antibody, other antibodies for cell surface molecules, including Toll-like receptor (TLR), Cytotoxic T-Lymphocyte Antigen 4 (CTLA-4), glucocorticoid- induced TNF receptor (GITR), OX40, and folate receptor 4 (FR4), have been developed [46]. However, an appropriate biomarker is needed to precisely monitor the responsiveness of the treated subjects. In the present survey, there are trends of CD8^+^ T cell/Treg correlating well with alterations of systemic effector cells after Treg depletion (Fig. 6B). Nonetheless, these ratios still will not reflect the real changes of cytokines (Fig. 6A) and antigen-specific immunity (Fig. 6C) when Tregs are modulated.

In conclusion, the imbalance between effective and suppressive immunities can be identified during disease progression. This phenomenon can be reversed by the depletion of immuno-suppressive Treg cells, especially the effective depletion. Tumor antigen-specific immunity may be enhanced when starting the depletion of Treg cells. The development of new strategies for effective depletion of Treg cells will become an important strategy for cancer immunotherapy.

## Supporting Information

Figure S1 **Different expressions of immune components in ascites of early- and advanced-stage ovarian cancer patients. (A) Expression of CD11b^+^CD33^+^Lin^−^ myeloid suppressor cells in TACs of early- and advanced-stage ovarian cancer patients.** (A1) Representative figures of flow cytometric analyses of myeloid suppressor cells in TACs. (A2) Percentages of myeloid suppressor cells in TACs. *Note*: The percentages of myeloid suppressor cells in TACs between early- and advanced-stage ovarian cancer patients were not significantly different. (**B**) Concentrations of various cytokines in ascites of ovarian cancer patients. B1, IL-5; B2, IL-9; B3, IL-12; B4, IL-13; B5, IL-17. *Note*: The concentrations of these cytokines in ascites between early- and advanced-stage ovarian cancers did not alter significantly.
(TIF)

Figure S2 **Representative figures of flow cytometric analyses of various kinds of local immune effectors in TACs of mice challenged with PBS or WF-3 tumor cells.**
(TIF)

## References

[1] Sakaguchi S (2004) Naturally arising CD4^+^ regulatory T cells for immunologic self-tolerance and negative control of immune response. Annu Rev Immunol 22: 531–532. 15032588 10.1146/annurev.immunol.21.120601.141122

[2] Sakaguchi S, Miyara M (2007) Natural regulatory T cells: mechanisms of suppression. Trends Mol Med 13: 108–116. 17257897 10.1016/j.molmed.2007.01.003

[3] Annacker O, Pimenta-Araujo R, Burlen-Defranoux O, Barbosa TC, Cumano A, et al (2001) CD4^+^ CD25^+^ T cells regulate the expansion of peripheral CD4 T cells through the production of IL-10. J Immunol 166: 3008–3018. 11207250 10.4049/jimmunol.166.5.3008

[4] Nakamura K, Kitani A, Fuss I, Pedersen A, Harada N, et al (2004) TGF-β 1 plays an important role in the mechanism of CD4^+^ CD25^+^ regulatory T cell activity in both humans and mice. J Immunol 172: 834–842. 14707053 10.4049/jimmunol.172.2.834

[5] Grossman WJ, Verbsky JW, Barchet W, Colonna M, Atkinson JP, et al (2004) Human T regulatory cells can use the perforin pathway to cause autologous target cell death. Immunity 21: 589–601. 15485635 10.1016/j.immuni.2004.09.002

[6] Ghiringhelli F, Ménard C, Terme M, Flament C, Taieb J, et al (2005) CD4^+^ CD25^+^ regulatory T cell inhibit natural killer cell functions in a transforming growth factor-β-dependent manner. J Exp Med 202: 1075–1085. 16230475 10.1084/jem.20051511 PMC2213209

[7] Baecher-Allan C, Anderson DE (2006) Regulatory cells and human cancer. Semin Cancer Biol 16: 98–105. 16378733 10.1016/j.semcancer.2005.11.003

[8] Curiel TJ, Coukos G, Zou L, Alvarez X, Cheng P, et al (2004) Specific recruitment of regulatory T cells in ovarian carcinoma fosters immune privilege and predicts reduced survival. Nat Med 10: 942–949. 15322536 10.1038/nm1093

[9] Wolf D, Wolf AM, Rumpold H, Fiegl H, Zeimet AG, et al (2005) The expression of the regulatory T cell-specific fork-head box transcription factor FoxP3 is associated with poor prognosis in ovarian cancer. Clin Cancer Res 11: 8326–8331. 16322292 10.1158/1078-0432.CCR-05-1244

[10] Leffers N, Gooden MJ, de Jong RA (2009) Prognostic significance of tumor-infiltrating T-lymphocytes in primary and metastatic lesions of advanced stage ovarian cancer. Cancer Immunol Immunothe 58: 449–459. 10.1007/s00262-008-0583-5 PMC11030692 18791714

[11] Agarwal R, Kaye SB (2003) Ovarian cancer: strategies for overcoming resistance to chemotherapy. Nat Rev Cancer 3: 502–516. 12835670 10.1038/nrc1123

[12] DiSaia PJ, Bloss JD (2003) Treatment of ovarian cancer: new strategies. Gynecol Oncol 90: S24–S32. 12928003 10.1016/s0090-8258(03)00341-x

[13] Pfleiderer A (1984) Diagnosis and staging of ovarian cancer. J Cancer Res Clin Oncol 107: 81–88. 6715399 10.1007/BF00399376 PMC12253817

[14] Gonzalez-Diego P, Lopez-Abente G, Pollan M, Ruiz M (2000) Time trends in ovarian cancer mortality in Europe (1955–1993): effect of age, birth cohort and period of death. Eur J Cancer 36: 816–824. 10.1016/s0959-8049(00)00184-2 10974630

[15] Boon T, Cerottini JC, Van den Eynde B, van der Bruggen P, Van Pel A (1994) Tumor antigens recognized by T lymphocytes. Annu Rev Immunol 12: 337–365. 8011285 10.1146/annurev.iy.12.040194.002005

[16] Zhang L, Conejo-Garcia JR, Katsaros D, Gimotty PA, Massobrio M, et al (2003) Intra-tumoral T cells, recurrence, and survival in epithelial ovarian cancer. N Engl J Med 348: 203–213. 12529460 10.1056/NEJMoa020177

[17] Tomsová M, Melichar B, Sedláková I, Steiner I (2008) Prognostic significance of CD3+ tumor-infiltrating lymphocytes in ovarian carcinoma. Gynecol Oncol 108: 415–420. 18037158 10.1016/j.ygyno.2007.10.016

[18] Chu CS, Kim SH, June CH, Coukos G (2008) Immunotherapy opportunities in ovarian cancer. Expert Rev Anticancer Ther 8: 243–257. 18279065 10.1586/14737140.8.2.243

[19] Cheng WF, Hung CF, Chai CY, Chen CA, Lee CN, et al (2007) Generation and characterization of an ascitogenic mesothelin-expressing tumor model. Cancer 110: 420–431. 17559144 10.1002/cncr.22781 PMC3181493

[20] International Federation of Gynecology and Obstetrics (1987) Changes in definitions of clinical staging for carcinoma of the cervix and ovary: International Federation of Gynecology and Obstetrics. Am J Obstet Gynecol 156: 263–264. 10681275

[21] Cheng WF, Hung CF, Hsu KF, Chai CY, He L, et al (2001) Enhancement of sindbis virus self-replicating RNA vaccine potency by targeting antigen to endosomal/lysosomal compartments. Hum Gene Ther 12: 235–252. 11177561 10.1089/10430340150218387

[22] Liao CW, Chen CA, Lee CN, Su YN, Chang MC, et al (2005) Fusion protein vaccine by domains of bacterial exotoxin linked with a tumor antigen generates potent immunologic responses and antitumor effects. Cancer Res 65: 9089–9098. 16204084 10.1158/0008-5472.CAN-05-0958

[23] Nakamura T, Yoshitani M, Rigby H, Fullwood NJ, Ito W, et al (2004) Sterilized, freeze-dried amniotic membrane: a useful substrate for ocular surface reconstruction. Invest Ophthalmol Vis Sci 45: 93–99. 14691159 10.1167/iovs.03-0752

[24] Roord ST, de Jager W, Boon L, Wulffraat N, Martens A, et al (2008) Autologous bone marrow transplantation in autoimmune arthritis restores immune homeostasis through CD4+CD25+Foxp3+ regulatory T cells. Blood 111: 5233–5241. 18256318 10.1182/blood-2007-12-128488

[25] Cheng WF, Hung CF, Chai CY, Hsu KF, He L, et al (2001) Enhancement of Sindbis virus self-replicating RNA vaccine potency by linkage of Mycobacterium tuberculosis heat shock protein 70 gene to an antigen gene. J Immunol 166: 6218–6226. 11342644 10.4049/jimmunol.166.10.6218

[26] Cheng WF, Lee CN, Su YN, Chang MC, Hsiao WC, et al (2005) Induction of human papillomavirus type 16-specific immunologic responses in a normal and an human papillomavirus-infected populations. Immunology 115: 136–149. 15819706 10.1111/j.1365-2567.2005.02126.x PMC1782130

[27] Imai H, Saio M, Nonaka K, Umemura N, Ouyang GF, et al (2007) Depletion of CD4+CD25+ regulatory T cells enhances interleukin-2-induced antitumor immunity in a mouse model of colon adenocarcinoma. Cancer Sci 98: 416–423. 17270031 10.1111/j.1349-7006.2006.00385.x PMC11158133

[28] Fahlén L, Read S, Gorelik L, Hurst SD, Coffman RL, et al (2005) T cells that cannot respond to TGF-beta escape control by CD4(+)CD25(+) regulatory T cells. J Exp Med 201: 737–746. 15753207 10.1084/jem.20040685 PMC2212836

[29] Li Q, Carr A, Ito F, Teitz-Tennenbaum S, Chang AE (2003) Polarization effects of 4-1BB during CD28 costimulation in generating tumor-reactive T cells for cancer immunotherapy. Cancer Res 63: 2546–2552. 12750278

[30] Workman CJ, Rice DS, Dugger KJ, Kurschner C, Vignali DA (2002) Phenotypic analysis of the murine CD4-related glycoprotein, CD223 (LAG-3). Eur J Immunol 32: 2255–2263. 12209638 10.1002/1521-4141(200208)32:8<2255::AID-IMMU2255>3.0.CO;2-A

[31] den Boer AT, van Mierlo GJ, Fransen MF, Melief CJ, Offringa R, et al (2005) CD4^+^ T cells are able to promote tumor growth through inhibition of tumor-specific CD8^+^ T cell responses in tumor-bearing hosts. Cancer Res 65: 6984–6989. 16061684 10.1158/0008-5472.CAN-04-3344

[32] Lawrence CW, Braciale TJ (2004) Activation, differentiation and migration of naive virus-specific CD8^+^ T cells during pulmonary influenza virus infection. J Immunol 173: 1209–1218. 15240712 10.4049/jimmunol.173.2.1209

[33] Golumbek PT, Lazenby AJ, Levitsky HI, Jaffee LM, Karasuyama H, et al (1991) Treatment of established renal cancer by tumor cells engineered to secrete interleukin-4. Science 254: 713–716. 1948050 10.1126/science.1948050

[34] Lejeune FJ (2002) Clinical use of TNF revisited: improving penetration of anti-cancer agents by increasing vascular permeability. J Clin Invest 110: 433–435. 12189235 10.1172/JCI16493 PMC150423

[35] Yigit R, Massuger LF, Figdor CG, Torensma R (2010) Ovarian cancer creates a suppressive micro-environment to escape immune elimination. Gynecol Oncol 117: 366–372. 20144842 10.1016/j.ygyno.2010.01.019

[36] Kryczek I, Wei S, Zhu G, Myers L, Mottram P, et al (2007) Relationship between B7-H4, regulatory T cells, and patient outcome in human ovarian carcinoma. Cancer Res 67: 8900–8905. 17875732 10.1158/0008-5472.CAN-07-1866

[37] Shankaran V, Ikeda H, Bruce AT, White JM, Swanson PE, et al (2001) IFN-gamma and lymphocytes prevent primary tumour development and shape tumour immunogenicity. Nature 410: 1107–1111. 11323675 10.1038/35074122

[38] Dunn GP, Bruce AT, Ikeda H, Old LJ, Schreiber RD (2002) Cancer immuno-editing: from immuno-surveillance to tumor escape. Nat Immunol 3: 991–998. 12407406 10.1038/ni1102-991

[39] Hallermalm K, Seki K, De Geer A, Motyka B, Bleackley RC, et al (2008) Modulation of the tumor cell phenotype by IFN-gamma results in resistance of uveal melanoma cells to granule-mediated lysis by cytotoxic lymphocytes. J Immunol 180: 3766–3774. 18322182 10.4049/jimmunol.180.6.3766

[40] Malmberg KJ, Levitsky V, Norell H, de Matos CT, Carlsten M, et al (2002) IFN-gamma protects short-term ovarian carcinoma cell lines from CTL lysis via a CD94/NKG2A-dependent mechanism. J Clin Invest 110: 1515–1523. 12438449 10.1172/JCI15564 PMC151808

[41] Wynn TA (2005) T(H)-17: a giant step from T(H)1 and T(H)2. Nat Immunol 6: 1069–1070. 16239919 10.1038/ni1105-1069

[42] Dong C (2006) Diversification of T-helper-cell lineages: finding the family root of IL-17-producing cells. Nat Rev Immunol 6: 329–333. 16557264 10.1038/nri1807

[43] Kryczek I, Banerjee M, Cheng P, Vatan L, Szeliga W, et al (2009) Phenotype, distribution, generation, and functional and clinical relevance of Th17 cells in the human tumor environments. Blood 114: 1141–1149. 19470694 10.1182/blood-2009-03-208249 PMC2723011

[44] Ioannides CG, Platsoucas CD, Rashed S, Wharton JT, Edwards CL, et al (1991) Tumor cytolysis by lymphocytes infiltrating ovarian malignant ascites. Cancer Res 51: 4257–4265. 1868446

[45] Sato E, Olson SH, Ahn J, Bundy B, Nishikawa H, et al (2005) Intra-epithelial CD8+ tumor-infiltrating lymphocytes and a high CD8+/regulatory T cell ratio are associated with favorable prognosis in ovarian cancer. Proc Natl Acad Sci USA 102: 18538–18543. 16344461 10.1073/pnas.0509182102 PMC1311741

[46] Nishikawa H, Sakaguchi S (2010) Regulatory T cells in tumor immunity. Int J Cancer 127: 759–767. 20518016 10.1002/ijc.25429

[47] Chen CA, Ho CM, Chang MC, Sun WZ, Chen YL, et al (2010) Metronomic chemotherapy enhances antitumor effects of cancer vaccine by depleting regulatory T lymphocytes and inhibiting tumor angiogenesis. Mol Ther 18: 1233–1243. 20372107 10.1038/mt.2010.34 PMC2889744

[48] Tsuji NM, Mizumachi K, Kurisaki J (2003) Antigen-specific, CD4^+^CD25^+^ regulatoryTcell clones induced in Peyer's patches. Int Immunol 15: 525–534. 12663682 10.1093/intimm/dxg051

[49] Freeman CM, Chiu BC, Stolberg VR, Hu J, Zeibecoglou K, et al (2005) CCR8 is expressed by antigen-elicited, IL-10-producing CD4^+^CD25^+^ T cells, which regulate Th2-mediatedgranuloma formation in mice. J Immunol 174: 1962–1970. 15699124 10.4049/jimmunol.174.4.1962 PMC1599789

[50] Larmonier N, Marron M, Zeng Y, Cantrell J, Romanoski A, et al (2007) Tumor-derived CD4(+)CD25(+) regulatoryTcell suppression of dendritic cell function involves TGF-h and IL-10. Cancer Immunol Immunother 56: 48–59. 16612596 10.1007/s00262-006-0160-8 PMC11030031

