# Supplementary figures and images for: Depletion of Regulatory T Lymphocytes Reverses the Imbalance between Pro- and Anti-Tumor Immunities via Enhancing Antigen-Specific T Cell Immune Responses
Source: PLoS One. 2012 Oct 17;7(10):e47190. doi: 10.1371/journal.pone.0047190 (PMC3474819; doi:10.1371/journal.pone.0047190)

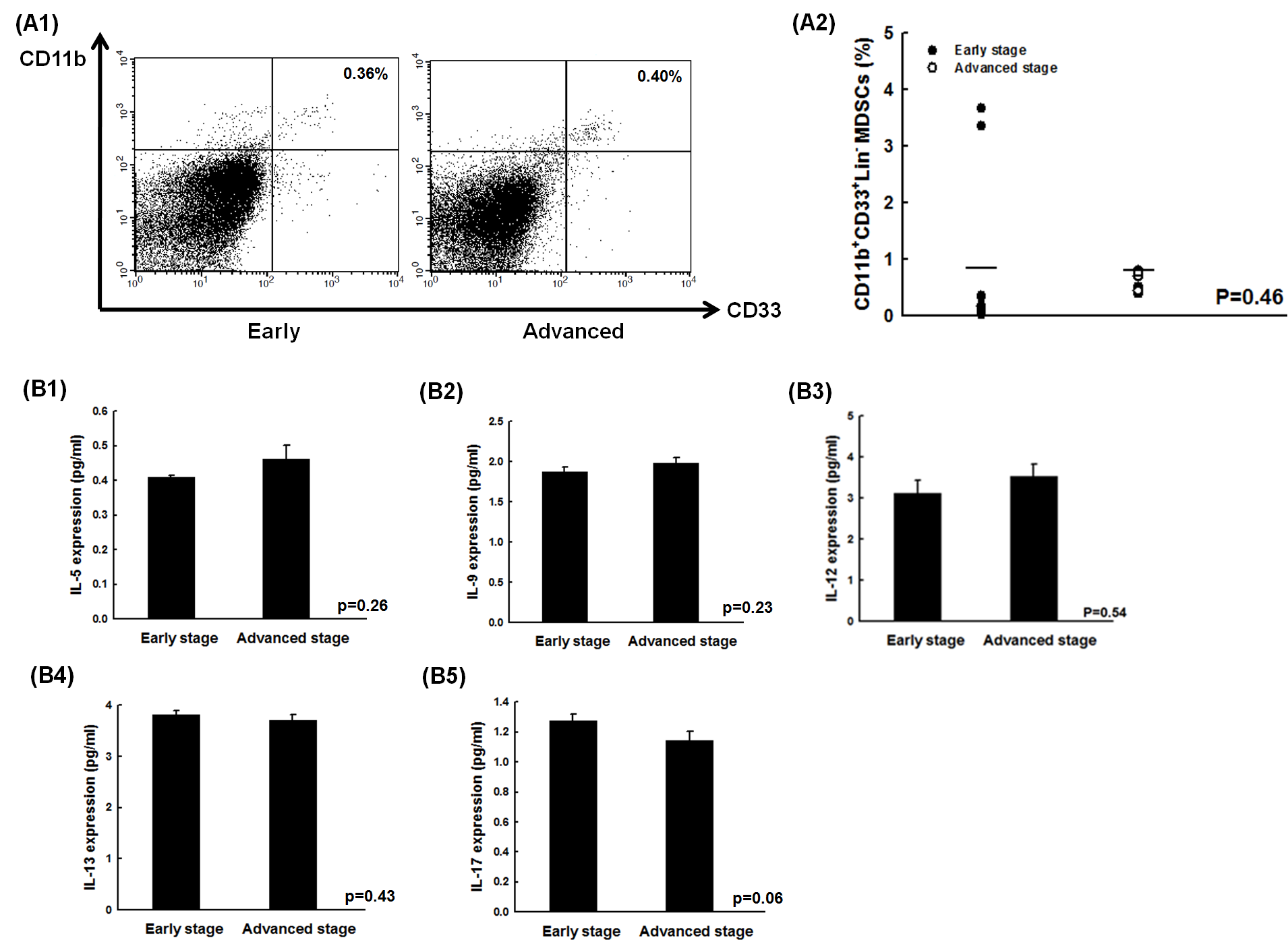

Supplement: Figure S1 — Different expressions of immune components in ascites of early- and advanced-stage ovarian cancer patients. (A) Expression of CD11b+CD33+Lin− myeloid suppressor cells in TACs of early- and advanced-stage ovarian cancer patients. (A1) Representative figures of flow cytometric analyses of myeloid suppressor cells in TACs. (A2) Percentages of myeloid suppressor cells in TACs. Note: The percentages of myeloid suppressor cells in TACs between early- and advanced-stage ovarian cancer patients were not significantly different. (B) Concentrations of various cytokines in ascites of ovarian cancer patients. B1, IL-5; B2, IL-9; B3, IL-12; B4, IL-13; B5, IL-17. Note: The concentrations of these cytokines in ascites between early- and advanced-stage ovarian cancers did not alter significantly. (TIF) [file pone.0047190.s001.tif]

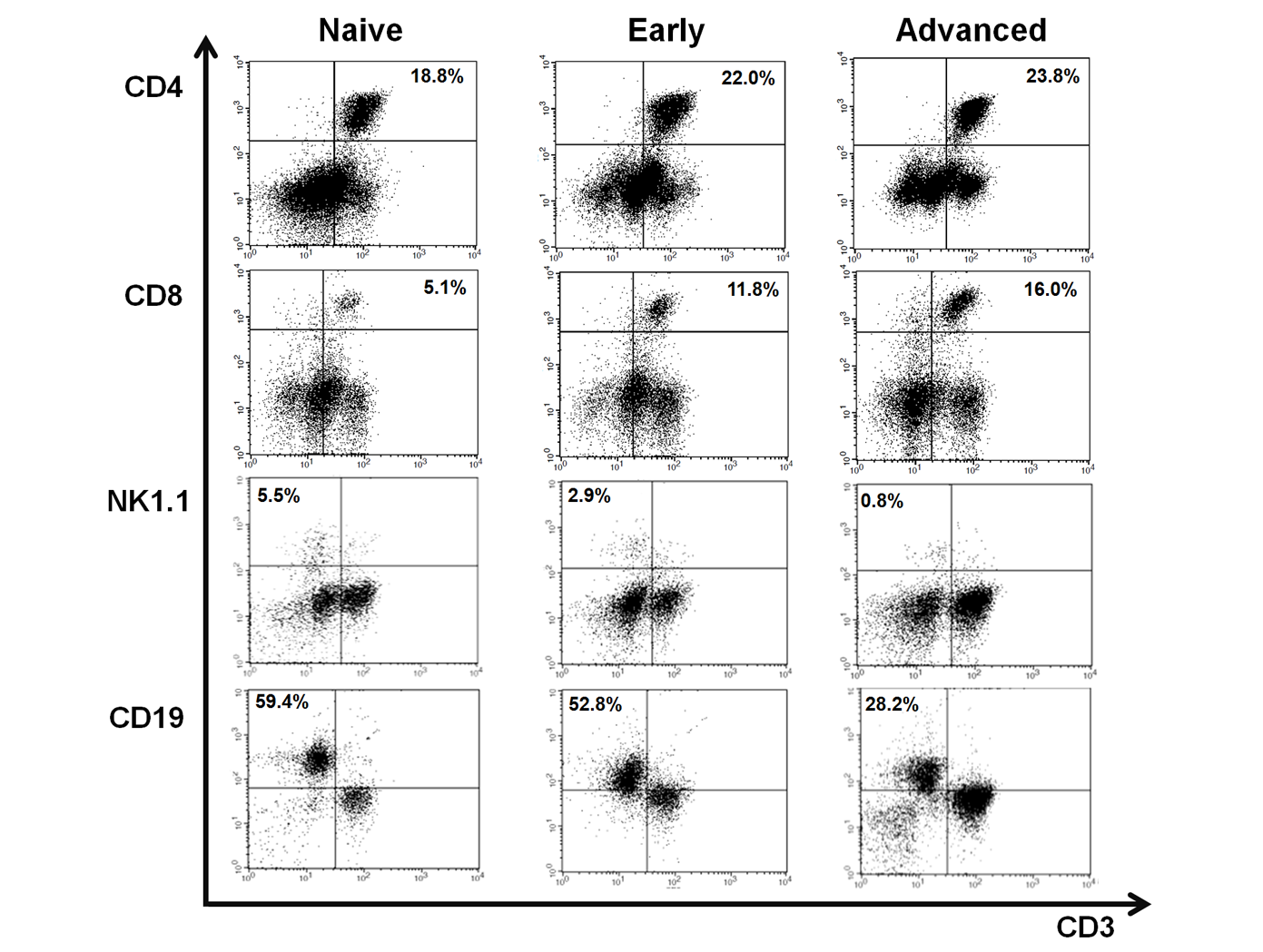

Supplement: Figure S2 — Representative figures of flow cytometric analyses of various kinds of local immune effectors in TACs of mice challenged with PBS or WF-3 tumor cells. (TIF) [file pone.0047190.s002.tif]
